# Myocardial Scar and Cardiac Biomarker Levels as Predictors of Mortality After Acute Myocardial Infarction: A CMR-Based Long-Term Study

**DOI:** 10.3390/diagnostics15243229

**Published:** 2025-12-17

**Authors:** Philipp Ruile, Johannes Brado, Klaus Kaier, Ramona Schmitt, Manuel Hein, Thomas Nührenberg, Hannah Billig, Franz-Josef Neumann, Dirk Westermann, Philipp Breitbart

**Affiliations:** 1 Department of Cardiology and Angiology, Medical Center—University of Freiburg, Faculty of Medicine, University of Freiburg, Südring 15, 79189 Bad Krozingen, Germany; philipp.ruile@uniklinik-freiburg.de (P.R.); manuel.hein@uniklinik-freiburg.de (M.H.); thomas.nuehrenberg@uniklinik-freiburg.de (T.N.); franz-josef.neumann@uniklinik-freiburg.de (F.-J.N.); dirk.westermann@uniklinik-freiburg.de (D.W.); 2 Institute of Medical Biometry and Statistics, Faculty of Medicine, University of Freiburg, Stefan-Meier-Straße 26, 79104 Freiburg im Breisgau, Germany; klaus.kaier@uniklinik-freiburg.de; 3 Department of Cardiology, University Hospital Bonn, Venusberg-Campus 1, 53127 Bonn, Germany; hannah.billig@ukbonn.de; 4 Cardioangiological Center Bethanien (CCB), Im Prüfling 23, 60389 Frankfurt, Germany

**Keywords:** myocardial infarction, cardiac magnetic resonance imaging, cardiac biomarkers

## Abstract

**Background/Objectives**: The extent of myocardial scar, visualized by late gadolinium enhancement (LGE) on cardiac magnetic resonance imaging (CMR), is associated with mortality after acute myocardial infarction (MI). However, data on optimal cardiac biomarker cut-off values (e.g., high-sensitivity cardiac troponin T, hs-cTnT) for predicting LGE remain limited. This study aimed to evaluate the predictive value of cardiac biomarkers for LGE and their influence on clinical outcomes. **Methods:** We included 597 patients who underwent CMR a median of 3 days [interquartile range (IQR) 2–4 days] after MI (407 STEMI and 190 NSTEMI patients), with a median follow-up period of 3.0 years [IQR 1.3–3.5 years]. **Results:** After adjusting for key variables, maximum cardiac biomarker levels were found to have the strongest correlation with the presence and extent of LGE (*p* < 0.001). LGE mass and LVEF were the most robust predictors of all-cause mortality (hazard ratio [CI] 1.464 [1.050–2.040], *p* = 0.025, Harrell’s C 0.812; 0.697 [0.491–0.990], *p* = 0.044, Harrell’s C 0.810, respectively). We determined a receiver operating characteristic (ROC) area under the curve (AUC) of 0.73 and an optimal cut-off of 53 g for LGE mass and mortality, with a maximum hs-cTnT cut-off of 7270 ng/L predicting this extent of LGE. **Conclusions:** In this large cohort of MI patients with three-year follow-up, cardiac biomarker levels showed a strong correlation with the extent of LGE. While absolute LGE mass was associated with mortality, its predictive value was comparable to that of CMR-derived LVEF. These findings should be interpreted cautiously, given the study’s observational design, and should be considered hypothesis-generating, underscoring the need for prospective validation.

## 1. Introduction

Despite significant advancements in interventional cardiology, myocardial infarction (MI) and its complications continue to be leading causes of mortality worldwide [1,2,3,4,5,6,7,8,9,10,11]. Previous studies have demonstrated that the infarct size (=scar size) assessed by cardiac magnetic resonance imaging (CMR) is strongly correlated with mortality [12,13,14,15,16]. In addition to infarct size, CMR allows for the assessment of microvascular obstruction (MVO), whose extent has been identified as an independent predictor of adverse outcomes, including increased mortality after MI, whereas the prognostic value of greyzone fibrosis appears to be more limited [17,18,19]. Accordingly, the 2023 ESC Guidelines for the management of acute coronary syndromes recognize the diagnostic capabilities of CMR, including direct visualization of infarcted myocardium and differentiation of myocardial scarring and viability from other forms of myocardial injury, while recommending its use mainly in cases of suboptimal echocardiographic image quality [20].

Furthermore, there is substantial evidence linking biomarkers with the extent of myocardial scar formation, although most of these studies have been conducted primarily in patients with ST-elevation myocardial infarction (STEMI) [21,22,23,24,25,26,27].

To date, there is a lack of data on cut-off values for cardiac biomarkers, such as high-sensitivity cardiac troponin T (hs-cTnT), creatine kinase (CK), and creatine kinase MB (CK-MB), in predicting myocardial scar formation. Additionally, the ability of these biomarkers to predict a prognostically relevant scar extent remains unclear. There are also scarce data regarding the predictive value of scar extent for mortality in patients with non-ST-elevation myocardial infarction (NSTEMI) [23].

This study aimed to determine which levels of routinely measured cardiac biomarkers predict the extent of prognostically relevant myocardial scar, as assessed by CMR, in patients with acute myocardial infarction (including both STEMI and NSTEMI).

## 2. Methods

This observational single-center data analysis included all patients who experienced acute MI (STEMI or NSTEMI with angiographically confirmed culprit lesions) between October 2008 and August 2023, and subsequently underwent CMR prior to discharge. Patients with a history of prior MI, chronic total coronary artery occlusion, or those with a suboptimal image quality were excluded from the study. In cases of STEMI, patients were directly transferred to the catheterization laboratory by emergency medical services, where immediate coronary angiography and intervention were performed. For NSTEMI cases, the timing of the procedure was determined by experienced interventional cardiologists certified by the German Cardiac Society.

Cardiac biomarkers were assessed at admission and at 8, 16, 24, and 48 h following percutaneous coronary intervention. In accordance with clinical practice and guideline recommendations during the study period, all patients without contraindications received standard post–myocardial infarction therapy, including ACE inhibitors or angiotensin receptor blockers and beta-blockers, which were administered in more than 95% of cases. Patients were followed up according to a standardized protocol, which included annual assessments for up to 8 years. Clinical and follow-up data were retrieved from our institutional database. Informed consent was obtained from all patients for the pseudonymized use of their clinical, procedural, and follow-up data at the time of their intervention. The study was conducted in accordance with the Declaration of Helsinki, and approved by the Ethics Committee of the Albert-Ludwigs-University Freiburg (protocol code 23-1559-S1-retro) on 27 Febrarury 2024.

### 2.1. Cardiac Magnetic Resonance Imaging

All CMR examinations were performed using a 3.0 Tesla scanner (Siemens Magnetom Skyra until 2020 and Siemens Magnetom Vida Fit from 2020 onwards, Siemens Healthineers, Forchheim, Germany). Patients were positioned in a supine position and imaged with a cardiac coil. Images were captured during end-expiratory breath-holds.

To assess cardiac function, retrospective ECG-gated steady-state free precession (SSFP) cine images were obtained in a short-axis (SAX) stack covering the left ventricle from base to apex, as well as in two-, three-, and four-chamber views. The parameters for SSFP cine imaging were as follows: echo time (TE) 1.4 ms, repetition time (TR) 2.9 ms, flip angle 60°, image resolution 1.5 × 1.5 × 8 mm, and slice gap 0 mm. Parallel imaging was not used to enhance the signal-to-noise ratio.

Late gadolinium enhancement (LGE) imaging was performed 10 to 15 min after injection of gadolinium-based contrast agents, using the same imaging planes as the cine images. LGE images were acquired with a phase-sensitive inversion-recovery sequence (TE 3.3 ms, TR 7.0 ms, inversion time (TI) 250–500 ms to nullify the myocardium, slice thickness 8 mm, no gap, matrix 256 × 192).

### 2.2. Image Analysis

Image analysis was performed using specialized post-processing workstations (syngo.via, Siemens Healthineers, Forchheim, Germany; CVI42, Circle Cardiovascular Imaging Inc., Calgary, AB, Canada) by two experienced readers (P.B. and P.R., each certified with the highest CMR qualification from the German Cardiac Society), who worked independently.

We semi-automatically assessed volume measurements, left ventricular ejection fraction (LVEF), stroke volume, cardiac index, and myocardial mass using SSFP cine images. For these analyses, the endocardial and epicardial borders of the left ventricle were manually delineated in all short-axis (SAX) slices at end-diastole and end-systole, with papillary muscles excluded from the myocardium.

We quantified LGE semi-automatically in short-axis images by applying signal intensity thresholds greater than five standard deviations above the mean signal intensity of remote myocardium. All automatically identified regions were visually inspected and adjusted as needed.

### 2.3. Statistical Analysis

Stata (StataCorp LLC, College Station, TX, USA, version 18) was used for all statistical analyses. Continuous variables are expressed as mean ± standard deviation (SD) or as median and interquartile range (IQR), and categorical variables as frequencies and percentages.

To compare two groups, the χ^2^-test was applied for categorical variables, Student’s *t*-test for continuous variables with normal distribution, and the Mann–Whitney U-test for continuous variables without normal distribution. The Kaplan–Meier method was used to visualize overall survival. Receiver operating characteristic (ROC) analyses were performed to determine the area under the curve (AUC) for predicting one-year survival using late gadolinium enhancement (LGE), with cardiac biomarker cut-off levels established using the Youden Index. Univariable and multivariable linear and Cox regression models were used to analyze LGE mass and overall survival, respectively. In order to assess the nonlinearity of the predictor LGE mass on overall survival, restricted cubic splines, with four knots chosen according to Harrells recommended percentiles, were used in the context of risk-adjusted linear and Cox regression models. Survival analyses were performed using all-cause mortality as the primary endpoint. Covariates for the multivariable Cox regression models (age, sex, infarct type) were selected a priori based on clinical relevance and established prognostic value. A *p*-value < 0.05 was considered statistically significant in all analyses.

## 3. Results

**Figure 1:**
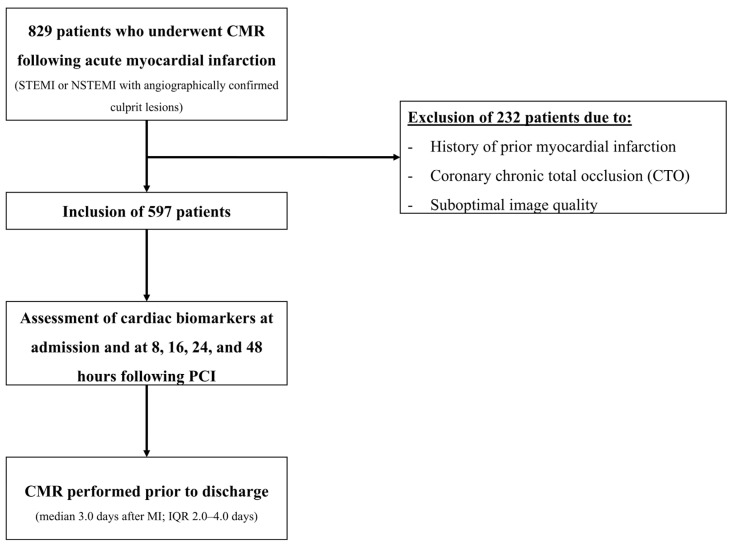
Study flowchart.

We screened 829 patients who underwent CMR following acute MI. Of these, 232 patients were excluded due to a history of prior MI, chronic total coronary artery occlusion, or suboptimal image quality (Figure 1).

The final cohort consisted of 597 patients (78.1% male, mean age 63.9 ± 11.7 years) with acute MI, including 407 patients (68.2%) with STEMI and 190 patients (31.8%) with NSTEMI. The most prevalent cardiovascular risk factors in the study population were dyslipidemia (*n* = 438, 73.4%), hypertension (*n* = 413, 69.2%), and smoking (*n* = 312, 52.3%). For 381 patients (63.8%), the time between chest pain onset and hospital admission was precisely documented. Most of these patients were admitted within 2 to 8 h of onset (*n* = 147; 38.6%) or within 2 h (*n* = 116; 19.4%).

Compared to NSTEMI patients, those with STEMI were younger (63.3 ± 11.9 vs. 65.4 ± 11.1 years, *p* = 0.035), had a lower prevalence of known coronary artery disease (8.8% vs. 18.0%, *p* = 0.002), and exhibited higher LDL cholesterol levels (143.9 ± 40.0 vs. 135.7 ± 42.6 mg/dL, *p* = 0.023).

The mean maximum levels of the cardiac biomarkers for the entire study cohort were as follows: hs-cTnT at 2440 ng/L [IQR 879, 5050 ng/L], CK at 979 U/L [IQR 398, 1925 U/L], and CK-MB at 110 U/L [IQR 47, 220 U/L]. Patients who experienced STEMI had significantly higher mean maximum levels for all biomarkers compared to those with NSTEMI: hs-cTnT (3270 vs. 1009 ng/L), CK (1285 vs. 400 U/L), and CK-MB (145 vs. 47 U/L), with *p* < 0.001 for each (Table 1).

Table 1 summarizes the main baseline characteristics, along with laboratory, coronary, and CMR findings, including a comparison between STEMI and NSTEMI patients. Sex-specific analyses are provided in Supplementary Table S1. Male patients in the cohort were younger, with a higher BMI and with lower LDL levels than women (*p* < 0.001, *p* = 0.002, *p* = 0.002).

### 3.1. CMR Findings

The mean time between MI and CMR was 3.0 days [IQR 2.0, 4.0 days], with no significant difference between STEMI and NSTEMI patients (*p* = 0.369). The mean LVEF at the time of CMR was 50.0 ± 10.0%, with lower values in STEMI patients compared to NSTEMI patients (48.8± 10.0% vs. 52.7 ± 9.5%, *p* < 0.001). LGE was present in 553 patients (92.6%), occurring in 388 STEMI patients (95.3%) and 165 NSTEMI patients (86.8%). The mean LGE mass for the entire cohort was 18.0 g [IQR 7.0, 34.0 g]. Male patients had a larger LGE mass than women (*p* < 0.001, Supplement Table S2).

There was no difference in the timing of MRI between patients with and without LGE (*p* = 0.915). A detailed comparison of baseline characteristics, laboratory results, coronary findings, and CMR outcomes between LGE-positive and LGE-negative patients is provided in Supplement Table S2. Age, sex, cardiovascular risk factors, known coronary artery disease (CAD), and LDL levels did not differ between the two groups.

However, patients with LGE had a significantly lower LVEF compared to those without LGE (49.4 ± 9.8% vs. 57.9 ± 9.0%, *p* < 0.001). Maximum levels of hs-cTnT, CK, and CK-MB were also significantly higher in LGE-positive patients (*p* < 0.001 for each). There was no significant difference in the infarct-related vessel between patients with and without LGE (*p* = 0.074).

### 3.2. Prediction of LGE

Regression analyses and assessments of predictive power were adjusted for key variables, including maximum cardiac biomarker levels, sex, age, infarction type, time from symptom onset to admission, infarct-related vessel, and high-sensitivity C-reactive protein (hs-CRP). Our analysis revealed that peak cardiac biomarker levels exhibited the strongest correlation with both the presence and extent of LGE (Table 2).

**Figure 2:**
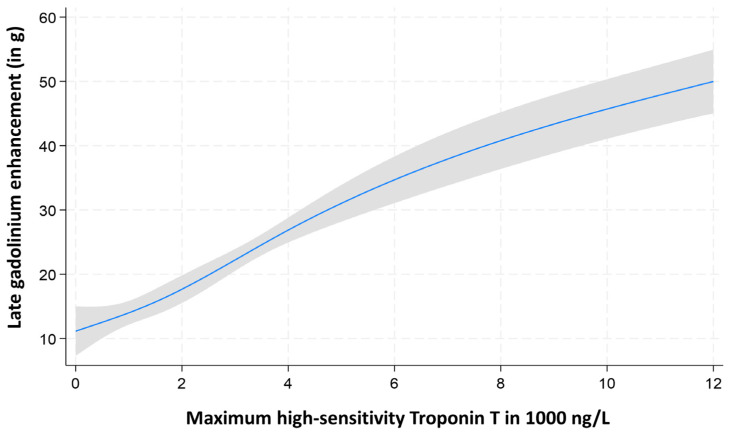
Assessment of the nonlinearity of maximum high-sensitivity cardiac troponin T for the prediction of late gadolinium enhancement mass.

**Figure 3:**
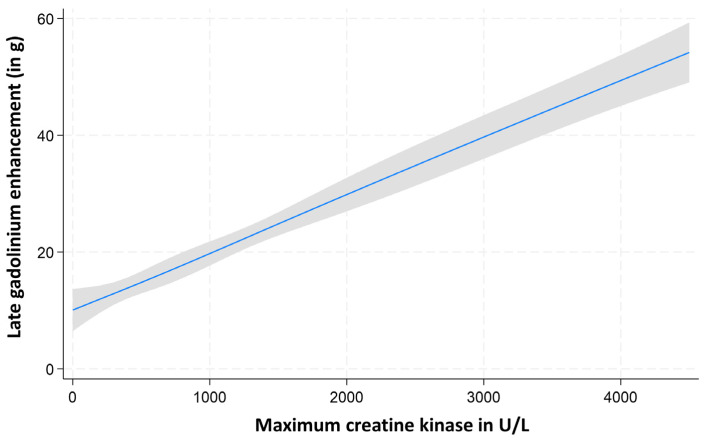
Assessment of the nonlinearity of maximum creatine kinase (CK) for the prediction of LGE mass.

**Figure 4:**
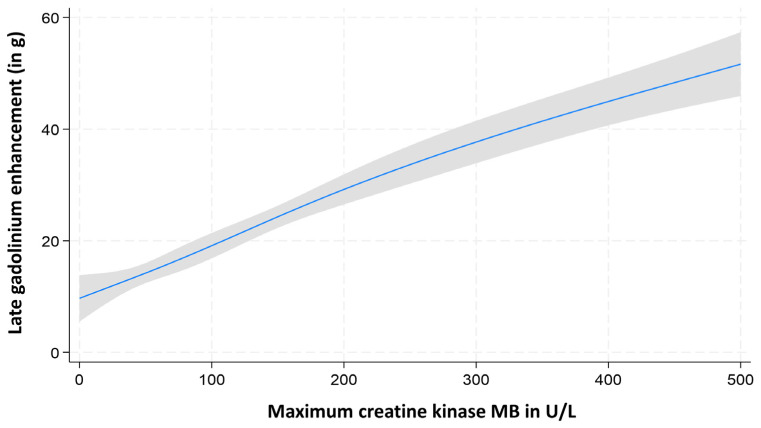
Assessment of nonlinearity of maximum creatine kinase MB (CK-MB) for prediction of LGE mass.

Multivariable linear regression models, adjusted for relevant covariates, confirmed that all cardiac biomarkers exhibited a linear relationship with LGE mass extent (Figure 2, Figure 3 and Figure 4).

### 3.3. Prediction of Survival (By MRI and Biomarkers)

**Figure 5:**
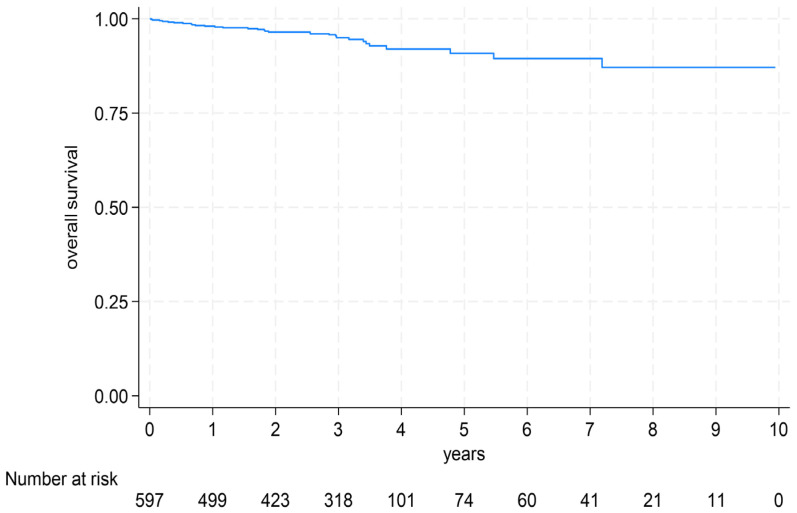
Overall survival in the follow-up period. The graph shows the overall survival of the study cohort in the follow-up period after the myocardial infarction.

The median follow-up duration was 3.0 years [IQR 1.3 to 3.5 years], with a mean of 3.0 ± 2.0 years. During this period, 32 patients (5.4% of the entire study cohort) died (Figure 5).

**Figure 6:**
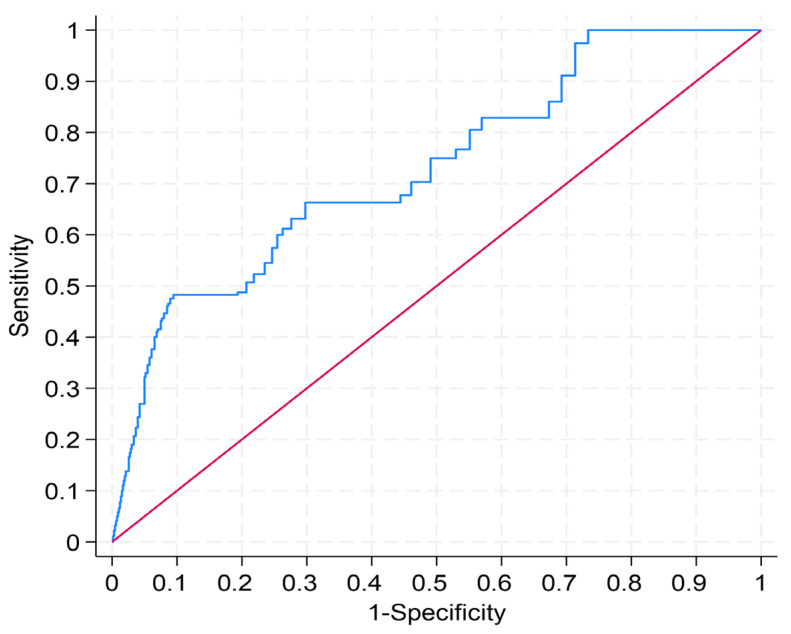
Receiver operating characteristic curve for survival data in relation to late gadolinium enhancement mass. Receiver operating characteristic curves for survival data were analyzed; one-year survival yielded an area under the curve (AUC) of 0.73 for late gadolinium enhancement mass. Using the Youden method, an optimal cut-off of 53 g was identified, yielding a sensitivity of 0.48 and a specificity of 0.91.

**Figure 7:**
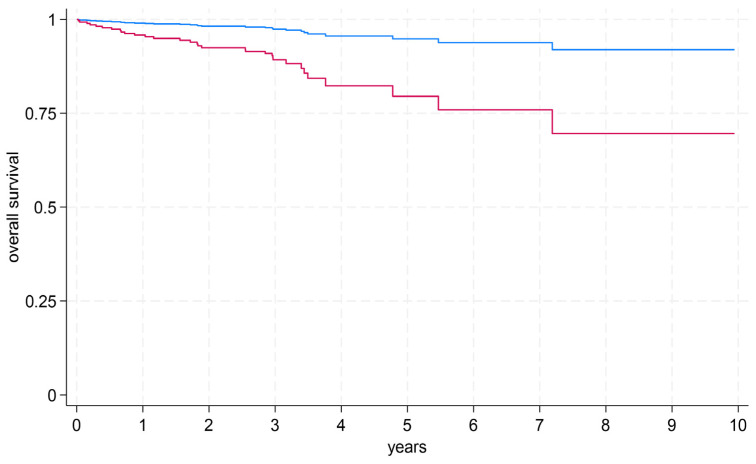
Overall survival after multivariable adjustment in the follow-up period for patients with LGE mass below (blue line) and above (red line) 53 g.

After adjusting for age, sex, and infarct type, absolute LGE mass (in g) and LVEF emerged as the strongest predictors of all-cause mortality (hazard ratio [CI] 1.464 [1.050–2.040], *p* = 0.025, Harrell’s C 0.812; 0.697 [0.491–0.990], *p* = 0.044, Harrell’s C 0.810, respectively) (Table 3). Receiver operating characteristic (ROC) curve analysis for one-year survival data yielded an area under the curve (AUC) of 0.73 for LGE mass. Using the Youden method, we identified 53 g as the optimal cut-off value, providing a sensitivity of 0.48 and a specificity of 0.91 for all-cause mortality (Figure 6). In multivariable Cox regression adjusted for age, sex, and infarct type, patients above this threshold had a markedly higher risk of all-cause mortality (hazard ratio 4.32, *p* = 0.052; Figure 7), although this association did not reach conventional statistical significance, likely owing to the limited number of events.

**Figure 8:**
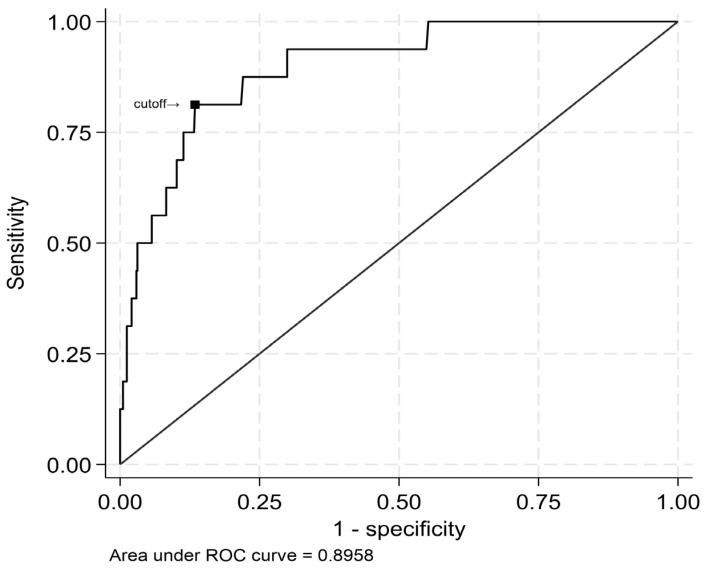
Receiver operating characteristic curve for survival data for identifying the ability of maximum levels of hs-cTnT to predict the former described cut-off value for LGE mass of 53 g. Based on the Youden method, an optimal cut-off was identified at a maximum hs-cTnT of 7270 ng/L to predict a LGE mass of at least 53 g. This maximum hs-cTnT cut-off yields a sensitivity of 0.81 and a specificity of 0.87.

ROC curve analysis was used to identify the ability of maximum levels of hs-cTnT to predict the previously described cut-off value for LGE mass of 53 g. Overall, maximum levels of hs-cTnT showed an excellent discrimination (AUC = 0.90, [95%CI 0.82–0.97]). Based on the Youden method, an optimal cut-off was identified at a maximum hs-cTnT 7270 ng/L to predict a LGE mass of at least 53 g. This maximum hs-cTnT cut-off leads to a sensitivity of 0.81 and a specificity of 0.87 for an LGE mass of at least 53 g (Figure 8).

## 4. Discussion

To our knowledge, this study represents one of the largest cohorts of patients undergoing CMR following acute MI, with comprehensive long-term follow-up.

The key findings are as follows:

• A strong correlation was observed between peak cardiac biomarker levels and both the presence and extent of myocardial scar tissue in patients with acute MI.
• Absolute LGE mass (in grams) and LVEF emerged as robust predictors of all-cause mortality with comparable predictive value.
• LGE mass of ≥53 g was strongly associated with elevated risk of all-cause mortality, and a peak hs-cTnT level of ≥7270 ng/L accurately predicted this extent of myocardial damage.

Following acute MI, precise quantification of myocardial injury is crucial for risk stratification and prognostication. However, due to resource constraints, routine CMR for all post-MI patients is not practical. Therefore, it is crucial to prioritize the use of this imaging modality for patients who are likely to derive the greatest benefit.

Our findings demonstrate a strong correlation between hs-cTnT levels and LGE-defined scar burden. These results align with the findings of Tiller et al., who reported a similar relationship between troponin levels and scar extent in a smaller cohort of STEMI patients [21]. Furthermore, a separate study, though limited in sample size, indicated a less pronounced correlation in NSTEMI patients [23].

There are conflicting data regarding the predictive value of infarct size or LGE percentage of the myocardium for clinical outcomes. Two studies have identified LGE extent as a predictor of major adverse cardiovascular events (MACE), independent of other factors such as ejection fraction, in patients with STEMI [12,15]. However, another large study in STEMI patients found no significant correlation between infarction size, expressed as LGE-percentage of myocardium, and outcomes [14]. In this context, our study provides clear incremental value by demonstrating that absolute LGE mass is a strong and clinically meaningful predictor of mortality in a large cohort of both STEMI and NSTEMI patients. This distinction is important, as absolute scar burden appears more closely linked to adverse outcomes and may therefore represent a more biologically plausible and clinically actionable metric than relative LGE percentages.

Data on optimal cut-off values for hs-cTnT levels in predicting clinically relevant scar extent are limited. Tiller et al. identified an hs-cTnT cut-off of 169.8 ng/L to predict an infarct size exceeding 19% of the myocardium (considered as a large infarction) in a cohort of 161 STEMI patients [21]. Another study reported a cut-off value of 3500 ng/L for ruling out severe left ventricular dysfunction following STEMI [24]. Our study adds further novelty by defining a substantially higher hs-cTnT threshold (7270 ng/L) that predicts an absolute scar mass ≥53 g. This cut-off correlates with a markedly increased risk of mortality. This biomarker-based threshold has the potential to serve as a clinically useful “gatekeeper,” enabling more selective deployment of CMR in patients who are most likely to benefit from advanced tissue characterization.

Notably, LVEF, assessed by CMR, shows the same level of predictive value in our study. These findings suggest that LGE-based assessment of myocardial damage is not mandatory for risk stratification in these patients. This is in line with a recently published large pooled analysis of more than 2000 patients, which found little additional value of LGE for predictive performance [28]. Beyond LVEF, even LVEF was not feasible for more accurate risk stratification. It is tempting to speculate that this difference is due to an echocardiographic evaluation of LVEF in this study, whereas our LVEF results were CMR-based.

Importantly, this should not be interpreted as diminishing the broader clinical value of CMR after MI. Beyond LGE quantification, CMR provides complementary and clinically relevant information that extends beyond echocardiography, including detailed assessment of myocardial viability, detection of mural thrombi that may be missed by echo, and precise characterization of microvascular obstruction (MVO). Furthermore, prior work from our group has demonstrated that extensive MVO is associated with adverse outcomes even in patients with an LVEF > 35% [17]. This finding has already influenced our clinical practice, where patients with pronounced MVO are equipped with a wearable cardioverter-defibrillator (LifeVest), and early arrhythmic events have been observed in this context.

We note that our study exclusively included patients with angiographically confirmed culprit lesions in STEMI and NSTEMI; therefore, diagnostic applications of CMR in MINOCA, where CMR plays a pivotal role [29], were outside the scope of this analysis. Nonetheless, in appropriately selected patients, CMR retains an important and expanding role beyond risk assessment based solely on LGE mass and LVEF.

### Limitations

The findings of our study should be interpreted in light of certain limitations. First, this was a single-center study, and CMR was performed at a single time point during the early post-infarction phase. This could potentially lead to an overestimation of LGE due to edema, as seen in other studies assessing early infarct size. However, the large sample size in our study may help mitigate this effect. In addition, although CMR was performed according to a predefined institutional protocol for all eligible patients, a few persistently unstable individuals who required prolonged intensive care or transfer to rehabilitation units could not be included. This may have introduced a minor selection bias.

Second, the proportion of patients with NSTEMI was relatively small, which limits the robustness of the data for this specific subgroup.

Finally, follow-up data were collected through a standardized protocol involving either phone contact or routine hospital visits, with no dedicated study-specific on-site follow-up visits for most patients.

## 5. Conclusions

Our study demonstrates a meaningful correlation between cardiac biomarker levels and myocardial damage as quantified by LGE on CMR. While absolute LGE mass showed prognostic relevance, its predictive value for mortality was comparable to that of CMR-derived LVEF. Given the observational design and inherent limitations of our study, these findings should be interpreted cautiously and considered hypothesis-generating. Rather than questioning the routine use of CMR, our results suggest that further prospective studies are needed to clarify the incremental prognostic role of LGE in this setting.

## Figures and Tables

**Table 1:** Baseline characteristics, as well as laboratory, coronary, and cardiac magnetic resonance imaging findings of the entire study cohort.

Characteristics	All Patients (*N* = 597)
Demographic	
Age—yr	63.9 ± 11.7
Male sex—no. (%)	466 (78.1)
Body mass index (IQR) †	27.1 (24.8–29.7)
Cardiovascular risk factors—no. (%)	
Hypertension	413 (69.2)
Dyslipidemia	438 (73.4)
Diabetes mellitus	113 (18.9)
Family predisposition	166 (27.8)
Nicotine abuse	312 (52.3)
Known CAD	70 (11.7)
Laboratory values	
Hemoglobin—g/dL	14.5 ± 4.0
LDL-C—mg/dL	141.2 ± 41.0
GFR—mL/min/1.73 m^2^ (IQR)	84.1 (67.8–97.1)
Maximum High-sensitivity Troponin T—ng/L (IQR)	2440.0 (879.0–5050.0)
Maximum CK—U/L (IQR)	979.0 (398.0–1925.0)
Maximum CK-MB—U/L (IQR)	110.0 (47.0–220.0)
Infarct-related vessel	
LAD	278 (46.6)
LCX	91 (15.2)
RCA	228 (38.2)
CMR findings	
Time between MI and CMR—days (IQR)	3.0 (2.0–4.0)
LGE—g (IQR)	18.0 (7.0–34.0)
LVEF—%	50.0 ± 10.0

Plus–minus values are means ± SD. For continuous variables, the median and interquartile range are presented for non-normally distributed variables. CAD denotes coronary artery disease, CK creatine kinase, CMR cardiac magnetic resonance imaging, GFR glomerular filtration rate, IQR interquartile range, LDL low-density lipoprotein cholesterol, LAD left anterior descending artery, LCX left circumflex coronary artery, LGE late gadolinium enhancement, LVEF left ventricular ejection fraction, MI myocardial infarction, RCA right coronary artery, and U units. † The body mass index is the weight in kilograms divided by the square of the height in meters.

**Table 2:** Results of univariable (models 1 to 3) and multivariable (models 4 to 6) linear regression models on the endpoint “late gadolinium enhancement mass in g”.

	1	2	3	4	5	6
Maximum High-sensitivity Troponin T—ng/L	0.003			0.003		
	[0.003,0.004]			[0.002,0.003]		
	(0.000)			(0.000)		
Maximum CK—U/L		0.010			0.010	
		[0.009,0.012]			[0.008,0.011]	
		(0.000)			(0.000)	
Maximum CK-MB—U/L			0.083			0.081
			[0.065,0.100]			[0.064,0.097]
			(0.000)			(0.000)
Sex				−8.229	−9.887	−11.036
				[−10.927,−5.530]	[−12.463,−7.310]	[−13.889,−8.182]
				(0.000)	(0.000)	(0.000)
Age				−0.193	−0.030	−0.084
				[−0.320,−0.066]	[−0.150,0.090]	[−0.215,0.048]
				(0.003)	(0.623)	(0.211)
Type of myocardial infarction				4.185	3.707	4.408
				[0.410,7.959]	[0.144,7.271]	[0.571,8.244]
				(0.030)	(0.041)	(0.024)
Time between symptom onset and admission < 2 h				−0.918	−0.634	−1.516
				[−6.104,4.267]	[−5.439,4.170]	[−6.684,3.652]
				(0.728)	(0.795)	(0.565)
Time between symptom onset and admission between 2 and 8 h				0.985	−0.437	−1.006
				[−4.824,6.794]	[−6.483,5.610]	[−7.335,5.323]
				(0.739)	(0.887)	(0.755)
Time between symptom onset and admission between 8 and 14 h				3.796	1.287	3.075
				[−4.746,12.338]	[−5.902,8.475]	[−5.093,11.244]
				(0.383)	(0.725)	(0.460)
Time between symptom onset and admission between >25 h				3.377	5.884	5.544
				[−3.947,10.701]	[−1.123,12.892]	[−1.590,12.678]
				(0.366)	(0.100)	(0.127)
Time between symptom onset and admission unknown				−1.346	−0.611	−1.110
				[−7.250,4.557]	[−6.010,4.788]	[−6.524,4.304]
				(0.654)	(0.824)	(0.687)
Infarct-related vessel—LAD				0.000	0.000	0.000
				[0.000,0.000]	[0.000,0.000]	[0.000,0.000]
				(.)	(.)	(.)
Infarct-related vessel—LCX				1.165	0.324	0.928
				[−3.420,5.749]	[−3.571,4.219]	[−3.451,5.306]
				(0.618)	(0.870)	(0.678)
Infarct-related vessel—RCA				−1.177	−1.597	−1.147
				[−4.361,2.007]	[−4.719,1.525]	[−4.381,2.087]
				(0.468)	(0.315)	(0.486)
Hs-CRP				1.825	2.266	2.146
				[0.678,2.972]	[1.148,3.384]	[1.075,3.216]
				(0.002)	(0.000)	(0.000)
Constant	11.935	9.619	10.949	37.578	29.288	24.076
	[9.961,13.909]	[7.531,11.708]	[8.280,13.617]	[13.067,62.089]	[1.673,56.904]	[−3.458,51.611]
	(0.000)	(0.000)	(0.000)	(0.003)	(0.038)	(0.086)
Observations	597	597	597	597	597	597

Coefficients; 95% confidence intervals in brackets; *p*-values in parentheses. Hs-CRP denotes high-sensitivity C-reactive protein, CK creatine kinase, LAD left anterior descending artery, LCX left circumflex coronary artery, and RCA right coronary artery.

**Table 3:** Results of univariable (models 1 to 6) and multivariable (models 7 to 12) Cox regression models on the endpoint overall mortality.

	1	2	3	4	5	6	7	8	9	10	11	12
LGE mass	1.267						1.464					
	[0.935,1.717]						[1.050,2.040]					
	(0.127)						(0.025)					
LGE proportion		1.203						1.260				
		[0.875,1.654]						[0.881,1.801]				
		(0.254)						(0.206)				
Maximum High-sensitivity Troponin T—ng/L			1.332						1.260			
			[0.974,1.822]						[0.912,1.740]			
			(0.073)						(0.161)			
Maximum CK—U/L				1.064						1.203		
				[0.755,1.499]						[0.843,1.717]		
				(0.722)						(0.308)		
Maximum CK-MB—U/L					1.001						1.031	
					[0.677,1.480]						[0.687,1.548]	
					(0.998)						(0.881)	
Left ventricular ejection fraction						0.686						0.697
						[0.488,0.966]						[0.491,0.990]
						(0.031)						(0.044)
Sex (=female)							1.141	1.021	1.006	0.986	0.987	1.025
							[0.519,2.510]	[0.472,2.209]	[0.465,2.173]	[0.458,2.126]	[0.457,2.132]	[0.476,2.210]
							(0.742)	(0.958)	(0.988)	(0.972)	(0.974)	(0.949)
Age							1.109	1.107	1.105	1.109	1.106	1.105
							[1.066,1.154]	[1.064,1.152]	[1.062,1.149]	[1.065,1.153]	[1.063,1.150]	[1.062,1.150]
							(0.000)	(0.000)	(0.000)	(0.000)	(0.000)	(0.000)
Type of myocardial infarction							1.167	1.227	1.228	1.299	1.414	1.189
							[0.532,2.561]	[0.553,2.726]	[0.556,2.712]	[0.594,2.840]	[0.646,3.097]	[0.548,2.580]
							(0.700)	(0.615)	(0.612)	(0.512)	(0.386)	(0.661)
Harrell’s C	0.630	0.620	0.541	0.504	0.483	0.655	0.812	0.795	0.778	0.782	0.776	0.810
Observations	597	597	597	597	597	597	597	597	597	597	597	597

Hazard Ratios; 95% confidence intervals in brackets; *p*-values in parentheses. CK denotes creatine kinase, and LGE late gadolinium enhancement.

## Data Availability

The raw data supporting the conclusions of this article will be made available by the authors on request.

## Supplementary Materials

The following supporting information can be downloaded at: https://www.mdpi.com/article/10.3390/diagnostics15243229/s1, Figure S1: Survival in relation to late gadolinium enhancement proportion in percent of total myocardial mass. Table S1: Baseline characteristics, as well as laboratory, coronary, and cardiac magnetic resonance imaging findings of the male and female subgroups. Table S2: Baseline characteristics, as well as laboratory, coronary, and cardiac magnetic resonance imaging (CMR) findings of patients with and without late gadolinium enhancement. Table S3: Baseline characteristics, as well as laboratory, coronary, and cardiac magnetic resonance imaging (CMR) findings of patients with and without all-cause mortality during follow-up.

## Abbreviations

CK	creatine kinase
CMR	cardiac magnetic resonance imaging
hs-cTnT	high-sensitivity cardiac Troponin T
IQR	Interquartile range
LGE	late gadolinium enhancement
LVEF	left ventricular ejection fraction
MACE	major adverse cardiovascular events
MI	myocardial infarction
NSTEMI	non-ST-elevation myocardial infarction
ROC	receiver operating characteristics
SAX	short axis stack
SD	standard deviation
SSFP	steady state free precision
STEMI	ST-elevation myocardial infarction
