# Supplementary material for: Myocardial Scar and Cardiac Biomarker Levels as Predictors of Mortality After Acute Myocardial Infarction: A CMR-Based Long-Term Study
Source: Diagnostics (Basel). 2025 Dec 17;15(24):3229. doi: 10.3390/diagnostics15243229 (PMC12731461; doi:10.3390/diagnostics15243229)
Supplement: Supplementary file 1 [file diagnostics-15-03229-s001.zip › diagnostics-3915115-supplementary.pdf]

Supplement Figure S1 – Survival in relation to late gadolinium enhancement proportion in percent of total myocardial mass.

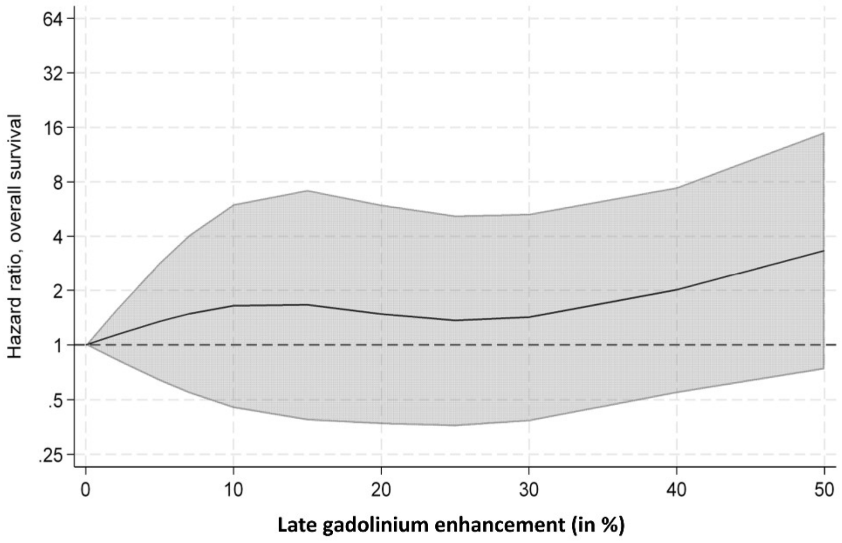

Supplement Table S1 – Baseline characteristics, as well as laboratory, coronary, and cardiac magnetic resonance imaging findings of the male and female subgroups.

Characteristics	Male (N=466)	Female (N=131)	P-Value
Demographic			
Age — yr	62.7±11.4	68.4±11.4	<0.001
Male sex — no. (%)			<0.001
Body-mass index (IQR)†	27.5 (25.0-29.8)	26.0 (23.2-28.9)	0.002
Cardiovascular risk factors — no. (%)			
Hypertension	314 (67.4)	99 (75.6)	0.092
Dyslipidemia	342 (73.4)	96 (73.3)	1
Diabetes mellitus	79 (17.0)	34 (26.0)	0.028
Family predisposition	134 (28.8)	32 (24.4)	0.386
Nicotine abuse	260 (55.8)	52 (39.7)	0.002
Known CAD	63 (13.5)	7 (5.3)	0.015
Laboratory values			
Hemoglobin — g/dL	14.9±4.4	13.3±1.3	<0.001
LDL-C — mg/dL	138.6±40.2	150.9±42.5	0.002
GFR — ml/min/1.73m^2 (IQR)	85.0 (70.3-98.2)	77.9 (61.0-90.9)	<0.001
Maximum High-sensitivity Troponin T — ng/L (IQR)	2530.0 (1003.5–5065.0)	2330.0 (752.5–4775.0)	0.272
Maximum CK — U/L (IQR)	987.0 (420.7–1938.5)	962.0 (329.0–1831.0)	0.587
Maximum CK-MB — U/L (IQR)	110.0 (47.0–210.0)	106.0 (44.5–270.5)	0.611
Type of myocardial infarction			0.270
STEMI	312 (67.0)	95 (72.5)	
NSTEMI	154 (33.0)	36 (27.5)	
Infarct-related vessel			0.348
LAD	212 (45.5)	66 (50.4)	
LCX	76 (16.3)	15 (11.5)	
RCA	178 (38.2)	50 (38.2)	
CMR findings			
Time between MI and CMR — days (IQR)	3.0 (2.0–4.0)	3.0 (2.0–4.0)	0.152
LGE — g (IQR)	20.0 (9.0–36.0)	11.0 (4.0–25.0)	<0.001
LVEF — %	49.7±9.9	51.3±10.5	0.120

Plus–minus values are means ±SD. For continuous variables, the median and interquartile range are presented for non-normally distributed variables. CAD denotes coronary artery disease, CK creatine kinase, CMR cardiac magnetic resonance imaging, GFR glomerular filtration rate, IQR interquartile range, LDL low-density lipoprotein cholesterol, LAD left anterior descending artery, LCX left circumflex coronary artery, LGE late gadolinium enhancement, LVEF left ventricular ejection fraction, MI myocardial infarction, NSTEMI non-ST-elevation myocardial infarction, RCA right coronary artery, STEMI ST-elevation myocardial infarction, and U units. † The body-mass index is the weight in kilograms divided by the square of the height in meters.

Supplement Table S2 – Baseline characteristics, as well as laboratory, coronary, and cardiac magnetic resonance imaging (CMR) findings of patients with and without late gadolinium enhancement.

**Characteristics**	**No LGE (N=44)**	**LGE (N=553)**	**P-Value**
Demographic			
Age — yr	64.9±11.5	63.9±11.7	0.586
Male sex — no. (%)	31 (70.5)	435 (78.7)	0.282
Body-mass index (IQR)†	27.1 (24.6-28.7)	27.2 (24.8-29.8)	0.383
Cardiovascular risk factors — no. (%)			
Hypertension	30 (68.2)	383 (69.3)	1
Dyslipidemia	30 (68.2)	408 (73.8)	0.528
Diabetes mellitus	10 (22.7)	103 (18.6)	0.639
Family predisposition	8 (18.2)	158 (28.6)	0.192
Nicotine abuse	23 (52.3)	289 (52.3)	1
Known CAD	5 (11.4)	65 (11.8)	1
Laboratory values			
Hemoglobin — g/dL	13.8±1.7	14.6±4.1	0.215
LDL-C — mg/dL	141.3±38.6	141.2±41.2	0.990
GFR — ml/min/1.73m^2 (IQR)	85.6 (64.9-94.4)	83.8 (68.0-97.3)	0.8
Maximum High-sensitivity Troponin T — ng/L (IQR)	218.0 (139.8–539.0)	2690.0 (1117.0–5310.0)	<0.001
Maximum CK — U/L (IQR)	164.5 (98.8–392.3)	1082.0 (488.0–2184.0)	<0.001
Maximum CK-MB — U/L (IQR)	26.0 (15.8–38.5)	120.0 (54.0–236.0)	<0.001
Type of myocardial infarction			<0.001
STEMI	19 (43.2)	388 (70.2)	
NSTEMI	25 (56.8)	165 (29.8)	
Infarct-related vessel			0.074
LAD	26 (59.1)	252 (45.6)	
LCX	2 (4.5)	89 (16.1)	
RCA	16 (36.4)	212 (38.3)	
CMR findings			
Time between MI and CMR — days (IQR)	3.0 (2.0–4.0)	3.0 (2.0–4.0)	0.915
LGE — g (IQR)	0	20.0 (10.0–35.0)	<0.001
LVEF — %	57.9±9.1	49.4±9.8	<0.001

Plus–minus values are means ±SD. For continuous variables, the median and interquartile range are presented for non-normally distributed variables. CAD denotes coronary artery disease, CK creatine kinase, CMR cardiac magnetic resonance imaging, GFR glomerular filtration rate, IQR interquartile range, LDL low-density lipoprotein cholesterol, LAD left anterior descending artery, LCX left circumflex coronary artery, LGE late gadolinium enhancement, LVEF left ventricular ejection fraction, MI myocardial infarction, NSTEMI non-ST-elevation myocardial infarction, RCA right coronary artery, STEMI ST-elevation myocardial infarction, and U units. † The body-mass index is the weight in kilograms divided by the square of the height in meters.

Supplement Table S3 – Baseline characteristics, as well as laboratory, coronary, and cardiac magnetic resonance imaging (CMR) findings of patients with and without all-cause mortality during follow-up.

Characteristics	No Death during follow-up (N=565)	Death during follow-up (N=32)	P-Value
Demographic			
Age — yr	63.4±11.5	74.5±9.0	<0.001
Male sex — no. (%)	444 (78.6)	22 (68.8)	0.277
Body-mass index (IQR)†	27.1 (24.7-29.7)	26.6 (25.3-29.6)	0.965
Cardiovascular risk factors — no. (%)			
Hypertension	389 (68.8)	24 (75.0)	0.592
Dyslipidemia	415 (73.5)	23 (71.9)	1.000
Diabetes mellitus	106 (18.8)	7 (21.9)	0.837
Family predisposition	162 (28.7)	4 (12.5)	0.074
Nicotine abuse	297 (52.6)	15 (46.9)	0.656
Known CAD	64 (11.3)	6 (18.8)	0.326
Laboratory values			
Hemoglobin — g/dL	14.6±4.1	14.1±1.6	0.549
LDL-C — mg/dL	142.2±40.9	123.7±40.1	0.014
GFR — ml/min/1.73m^2 (IQR)	84.4 (69.2.9-97.4)	66.0 (52.1-85.7)	<0.001
Maximum High-sensitivity Troponin T — ng/L (IQR)	2450.0 (883.0–4990.0)	2235.0 (820.8–5837.5)	0.865
Maximum CK — U/L (IQR)	990.0 (399.0–1955.0)	806.5 (303.8-1489.0)	0.266
Maximum CK-MB — U/L (IQR)	111.0 (47.0–229.0)	94.0 (41.8–128.8)	0.144
Type of myocardial infarction			1.000
STEMI	385 (68.1)	22 (68.8)	
NSTEMI	180 (31.9)	10 (31.2)	
Infarct-related vessel			0.738
LAD	261 (46.2)	17 (53.1)	
LCX	87 (15.4)	4 (12.5)	
RCA	217 (38.4)	11 (34.4)	
CMR findings			
Time between MI and CMR — days (IQR)	3.0 (2.0–4.0)	3.0 (3.0–5.0)	0.004
LGE — g (IQR)	18.0 (7.0-34.0)	19.0 (11.0–34.3)	0.311
LVEF — %	50.3±9.8	45.5±13.9	0.015

Plus–minus values are means ±SD. For continuous variables, the median and interquartile range are presented for non-normally distributed variables. CAD denotes coronary artery disease, CK creatine kinase, CMR cardiac magnetic resonance imaging, GFR glomerular filtration rate, IQR interquartile range, LDL low-density lipoprotein cholesterol, LAD left anterior descending artery, LCX left circumflex coronary artery, LGE late gadolinium enhancement, LVEF left ventricular ejection fraction, MI myocardial infarction, NSTEMI non-ST-elevation myocardial infarction, RCA right coronary artery, STEMI ST-elevation myocardial infarction, and U units.

† The body-mass index is the weight in kilograms divided by the square of the height in meters.
